# Reading Comprehension in Both Spanish and English as a Foreign Language by High School Spanish Students

**DOI:** 10.3389/fpsyg.2021.789207

**Published:** 2022-01-17

**Authors:** Elena Cueva, Marta Álvarez-Cañizo, Paz Suárez-Coalla

**Affiliations:** ^1^Department of Psychology, University of Oviedo, Oviedo, Spain; ^2^Department of Psychology, University of Valladolid, Valladolid, Spain

**Keywords:** Spanish, secondary students, EFL, reading comprehension, morphology, syntax, vocabulary

## Abstract

Several studies have highlighted that reading comprehension is determined by different linguistic skills: semantics, syntax, and morphology, in addition to one’s own competence in reading fluency (accuracy, speed, and prosody). On the other hand, according to the Linguistic Interdependence Hypothesis, linguistic skills developed in one’s own native language (L1) facilitate the development of these skills in a second one (L2). In this study, we wanted to explore the linguistic abilities that determine reading comprehension in Spanish (L1) and in English (L2) in Secondary Education students. To do this, 73 Secondary Education Students (1st and 3rd year) participated in this study. The students carried out a battery of tasks in English and Spanish, all of them related to reading comprehension (expository text) and different linguistic skills, which included syntactic awareness tasks, synonymy judgment tasks (vocabulary), and morphological awareness tasks. The results indicated a positive correlation between linguistic competencies in both languages (indicating a transfer effect between languages), which were determined by school year, with a lower performance in the 1st year than in the 3rd year. Moreover, we found more skills with correlations in English reading comprehension than in Spanish. Finally, reading comprehension in L1 was mainly explained English reading comprehension, while English reading comprehension was predicted by grade, and syntactic awareness, as well as Spanish reading comprehension. This could be explained by the different levels of exposure to L1 and L2 of sample subjects, as the linguistic variables have different influences on the reading comprehension of both languages.

## Introduction

Reading comprehension skills are a requirement to be successful in the academic, as well as professional realms of life (García and Cain, 2014). Furthermore, in our contemporary and global society, it is not sufficient to understand native language (L1) texts, it is also necessary to achieve reading proficiency in other languages. Specifically, English is the most used language in both work and study environments, therefore being taught as a second language (L2) in many countries where numerous children also follow bilingual programs in schools. In this context, studies about reading comprehension in L1 and L2 are of considerable relevance, as reading comprehension sometimes supposes an academic difficulty for L2 students, with lower language levels than monolingual peers (Low and Siegel, 2005). Some students learning in English (L2) might be at a disadvantage due to the lack of language development.

In Spain, children start to learn English in schools at a very early age and many of them follow bilingual or semi-bilingual programs from their 1st grade (6 years). They study some subjects in English and have English textbooks. Given that Spanish and English don’t share an origin, English being a Germanic opaque language, while Spanish is a Romance transparent one, having to read and learn in English could pose an additional challenge for Spanish children. Furthermore, although there is a great semantic correspondence between the concepts in Spanish and English (Vivas et al., 2020), there are many other differences between the languages on a morphological level (e.g., absence of gender in nouns or few conjugations of verbs in English) and syntax (e.g., in English there exists a mandatory use of the subject in sentences, unalterable order of words, use of simple negation versus double Spanish negation) (Valenzuela, 2002). This is an additional difficulty when it comes to the acquisition of this new language for Spanish children.

In addition, it is necessary to underline the low exposure to English, compared to Spanish, that children receive before the formal reading instruction begins. Most frequently, if they are in school, from the age of 3 (in Spain, schooling is not compulsory until the age of 6) exposure to English begins, averaging around 2 h a week. With the commencement of primary education (+6 years of age), the teaching of English is carried out in a more formal and academic manner. Students receive approximately 4 weekly hours of English classes. Moreover, there are certain bilingual schools where approximately half of the subjects are taught in English (e.g., science, music, and arts). When students complete their secondary education (two final years in addition to mandatory secondary school, 18 years old) it is assumed that they have reached an A2 level of Common European Framework of Reference (CERF) in the different competencies (comprehension and expression, both oral and written) of English. In the case of secondary students with a bilingual itinerary, the level would be B1or B1+ (CERF). Spanish children suffer the highly demanding situation of learning in the English language while simultaneously developing oral and reading proficiency. This condition could be affecting the development of linguistic competencies, and therefore, different language skills might be contributing to reading comprehension in Spanish and/or English.

On the other hand, regarding language proficiency, previous studies showed that language skills transfer across languages (Cummins, 1979; August and Shanahan, 2006). In other words, reading abilities in L1 might be transferred to L2 reading (D’Angelo and Chen, 2017; Tong et al., 2018). D’Angelo and Chen (2017) explored reading comprehension in English (native language) and French (L2). Three groups of comprehenders were identified (poor, average, and good) based on English reading performance. They found that poor comprehenders showed similar language characteristics both in L1 and L2. Similarly, Tong et al. (2018) reported the co-occurrence of reading comprehension difficulty in L1 Chinese and L2 English. Chinese–English L2 learners (10 years old) that manifested problems in L1 Chinese reading comprehension are likely to show low performance in L2 English reading comprehension. These results suggest that the comprehension skills developed in one language will facilitate reading comprehension in another language and reading comprehension profiles will be similar in both L1 and L2 languages.

In this field of study, over the last few years, research about reading comprehension in monolingual and bilingual populations has increased considerably (Choi et al., 2017; D’Angelo and Chen, 2017; Mackay et al., 2017; Spencer and Wagner, 2017; D’Angelo et al., 2020; Zhang et al., 2020; Zhao et al., 2021).

### Reading Comprehension

Reading comprehension is a universal process that consists of eliciting and conjuring meaning through interaction and involvement with written language (McNamara and Magliano, 2009). Reading comprehension is considered a very complex process, involving several abilities for the acquisition of significance from a written text (Kirby, 2007). However, the Simple View of Reading (SVR) states that reading comprehension depends on decoding skills and language comprehension (Hoover and Gough, 1990; Gottardo and Mueller, 2009), so some processes are not specific for reading comprehension. Furthermore, decoding skills improve with age and the influence on reading comprehension diminishes, while the effects of other skills such as vocabulary, syntax, and morphology remain, apart from inference skills, working memory, and monitoring (Hannon and Daneman, 2001; Perfetti and Hart, 2001; Perfetti et al., 2013; Landi and Ryherd, 2017). Although in recent years there have been quite a few studies on the components of language that contribute to reading comprehension in monolingual and bilingual children, little is known about whether reading comprehension skills are manifested similarly in L1 and L2 (and the components contributing to successful reading comprehension in English) for Spanish adolescents in a bilingual school context, where they study 50% of subjects in English, whilst the language of the community is Spanish.

### Vocabulary

The term vocabulary refers to the set of words that a person knows or uses, as well as to the words of a specific language (Hornby, 2006). It is presumed to be one of the most crucial language skills contributing to reading competence (National Reading Panel and National Institute of Child Health and Human Development (US), 2000; Fernandes et al., 2017; Sparapani et al., 2018; Quinn et al., 2020). It constitutes a pillar essential to reading success and progress (Lonigan, 2006; Dickinson et al., 2010). Likewise, it has been reported that children with poor comprehension around 9 years of age exhibit low levels of vocabulary (Nation et al., 2007; Ricketts et al., 2007; Hock et al., 2009). These deficits, not always evident, would limit the ability to understand a text with unfamiliar words. However, the impact of vocabulary on reading comprehension appears to depend on age (Protopapas et al., 2007). Protopapas et al. (2007) supported the idea that vocabulary becomes more important around 7–10 years of age, once word decoding is automated, results that coincide with those found in other studies (Hock et al., 2009). What’s more, it seems that in skilled readers there is a bidirectional relationship between vocabulary and reading comprehension (Quinn et al., 2020), signaling that vocabulary is a leading indicator of change in reading comprehension, and reading comprehension is a leading indicator of change in vocabulary.

Regarding L2 reading comprehension, several studies suggested that vocabulary, among other skills, determines its development in L2 (Lesaux and Kieffer, 2010; Li and Kirby, 2014; D’Angelo et al., 2017; Tong et al., 2018). However, Burgoyne et al. (2011) found that vocabulary was a predictor of English reading comprehension for 4th-grade bilingual children whose first language was of South-Asian origin, but not for those of their monolingual peers. It suggests that the contributions of language skills to reading comprehension could also depend, to a certain extent, on language exposure.

### Morphological Awareness

Morphological awareness is defined as the ability to manipulate morphemes and the structure of words (Kuo and Anderson, 2006). This metalinguistic consciousness, especially considering derivational morphology, continues to develop throughout schooling (Casalis and Louis-Alexandre, 2000), and it is important to achieve word meanings, in turn then favoring reading comprehension (McBride-Chang et al., 2003; Deacon and Kirby, 2004; Cain and Oakhill, 2006; Guo et al., 2011; Jeon and Yamashita, 2014; Tong et al., 2014; D’Alessio et al., 2019; Zhang et al., 2020; Kotzer et al., 2021; Li et al., 2021).

Regarding monolinguals, Carlisle (2000) reported that morphological awareness tasks contributed to text comprehension at both 3rd and 5th grades, but with a stronger effect for older children than for younger ones. Similarly, morphological awareness also appears to benefit reading comprehension, independent from word decoding, in 4th-grade Spanish-speaking children (D’Alessio et al., 2019). In addition, native English speakers seem to rely on morphology to infer the meaning of the new words encountered while reading (Crosson and McKeown, 2016), hence supposing an advantage to reading comprehension.

Concerning children who received education in L2, Lipka and Siegel (2012) found that English L2 poor comprehenders (7th grade) had lower scores in morphological awareness than good comprehenders. Similarly, in a study about children with English L1 and French L2 (10-to-11-year-old), French morphological awareness differentiated bilingual poor from good comprehenders, supporting the proposal that morphological awareness impacts reading comprehension when some language levels are achieved (D’Angelo and Chen, 2017). Recently, an interesting study addressed the role of (English) language proficiency (native, fluent, and limited proficiency) and morphological competence as beneficial for reading comprehension (Zhang et al., 2020). However, the contribution of morphological awareness to reading comprehension seems to be dependent on English proficiency, as participants with a higher English proficiency (native speakers and fluent levels) were better at taking advantage of morphological information to infer word meanings than participants with lower English levels (Zhang et al., 2020).

### Syntax Awareness

Syntactic awareness is the ability to reflect on grammar rules and to manipulate the grammatical structure of sentences in a language (Gombert, 1992). This ability to manipulate the syntactic structure of spoken language is generally considered related to reading development via its contribution to reading comprehension (Paris and Landauer, 1982; Bowey, 1986) and to word recognition (Tunmer et al., 1987; Tunmer and Hoover, 1992). Several reading models considered syntactic awareness as an important skill to achieve reading comprehension include: Simple View of Reading (Gough and Tunmer, 1986; Hoover and Gough, 1990), the Triangle Model (Seidenberg and McClelland, 1989; Bishop and Snowling, 2004) or the Reading Systems Framework (Perfetti, 1999; Perfetti et al., 2008; Perfetti and Stafura, 2014). Syntactic awareness is important for reading success, as it allows the anticipation of syntactic categories and the inference of which word class will follow (Tunmer and Bowey, 1984; Bishop and Snowling, 2004).

Some studies carried out with monolinguals found that poor comprehenders also have syntactic weaknesses. In a study with English fourth graders, Adlof and Catts (2015) found that poor comprehenders also had problems in some syntactic constructions as be-do questions in an orally grammatical judgment task. These findings are in agreement with other studies that relate poor comprehension to grammatical difficulties (Tong et al., 2018; Guo et al., 2020; Li et al., 2021).

Focusing on bilingual studies, the most thoroughly investigated issue is whether there exists a transfer skill between languages. In this sense, it has been shown that the syntax of Chinese–English elementary school children had an influence and predicted reading comprehension (Chik et al., 2012; Yeung et al., 2012; Siu and Ho, 2015). Moreover, in Chinese–English children, syntactic awareness improved from first to second grade in both L1 and L2; and L1 syntactic awareness predicted L2 reading comprehension 1 year later (Siu and Ho, 2020). Similar results were found in studies with Spanish-French children where L2 text comprehension was explained by L1 text comprehension and L1 syntactic awareness (Lefrançois and Armand, 2003). On top of that, for Spanish primary school students with English as a second language, findings showed that both syntax and morphology in oral language predicted levels of reading comprehension (Gottardo et al., 2018).

In conclusion, studies about comprehension in monolinguals and bilinguals reported that several linguistic skills contribute to reading comprehension. However, the contribution of different skills appears to vary depending on age or exposure to the language.

### The Current Study

The present study aims to explore the development of Spanish and English competence (vocabulary, morphology, syntaxis, and reading comprehension) of Spanish secondary school children (1st and 3rd grade) and the contribution of said abilities to reading comprehension in both languages, in absence of poor reading decoding. We are interested in students with adequate word-reading skills, so comprehension differences could not be attributed to decoding performance. In addition, it should be highlighted that these participants were native Spanish speakers receiving a Spanish–English bilingual education, so participants differed from immigrant children in English monolingual schools.

According to the language skills transference across languages theory, we hypothesized relationships between languages in different tasks; however, considering language-specific factors (such as exposure or practice) and the age of the participants, we expected differences in the contribution of linguistic skills to reading comprehension in Spanish and English.

## Methodology

### Participants

Seventy-three students participated in this study from the 1st (24 girls and 20 boys; *M*_*age*_ = 12.93, *SD* = 0.25), and 3rd grades of secondary school, equivalent to seventh and ninth grade, respectively, in the American and British education systems (20 girls and 9 boys; *M*_*age*_ = 14.81, *SD* = 0.25). The difference in the number of participants in each course may be due to greater involvement and interest in carrying out voluntary tasks in younger ages. Participants were recruited from two Spanish–English bilingual secondary schools in Asturias (Spain). Participants have been exposed to English from the beginning of preschool, around 3 years old. At the end of first grade, they have reached an A2 English level, although some may reach a B1; while at the end of third grade they are expected to have got a B1 level, although some students may have reached a B2. To teach English reading, instructors primarily employed a global method – introducing meaning, pronunciation, and spelling at the same time. At this point in time, children received 4 h of English language lessons per week, and they follow (from 1st grade of primary school) a Content and Language Integrated Learning methodology (CLIL; de Martínez Agudo, 2019), with 50% of subjects being taught in the English language.

All participants had Spanish as their first language and belonged to a middle-class socioeconomic status. None of them had developmental, behavioral, or cognitive issues, as 12 students with learning and academic difficulties were excluded from the study. Teachers confirmed that the schooling of all participants had been developed without suffering remarkable incidents and they had not retaken a year of studies. In addition, 5 participants were also removed for not completing the tasks and 3 were considered outliers because of their performance.

### Tasks

The present study consisted of four linguistic tasks in both Spanish and English languages:

(a) *Synonym judgment task* (Spanish and English versions). Thirty-two pairs of words were constructed, for which participants had to decide whether the two items of the pair had a similar meaning (e.g., courage-bravery [valor-valentía] and historieta-cuento [tale-story]). Although other semantic tasks could have been used, this task was selected granted its effectiveness, as seen in previous studies (D’Angelo and Chen, 2017). The English stimuli were selected according to their lexical frequency (Kuperman et al., 2012), and Spanish words were selected following their lexical frequency from B-pal (Davis and Perea, 2005). Considering the thirty-two pairs of words, the Cronbach’s alpha coefficient was 0.50 for the Spanish task and 0.71 for the English task, so we dropped some items to increase reliability. After dropping 8 items for each language, the Cronbach’s alpha coefficient was 0.62 for the Spanish task and 0.75 for the English task. The final Spanish task had a total of 10 pairs of not similar words and 14 pairs of similar words [*M*_*similar list*_ = 30.27, *SD* = 33.65; *M*_*different list*_ = 23.05, *SD* = 41.58; *t*(46) = 0.664, *p* = 0.51]. Besides, syllable count was similar in both lists [*M*_*similar list*_ = 3.25, *SD* = 1.04; *M*_*different list*_ = 3.2, *SD* = 0.69; *t*(46) = 0.815, *p* = 0.425]. Moreover, English task had 14 pairs of similar words and 10 pairs of not similar words, also with a similar lexical frequency [*M*_*similar list*_ = 23.76, *SD* = 24.14; *M*_*different list*_ = 22.87, *SD* = 23.07; *t*(46) = 0.129, *p* = 0.89] and syllabic length [*M*_*similar list*_ = 2.25, *SD* = 1.02; *M*_*different list*_ = 1.71, *SD* = 0.54; *t*(46) = 1.76, *p* = 0.09]. Therefore, the maximum score in each language task was 24, one point for each of the items (pair of words) correctly answered.

(b) *Syntactic judgment task*. This consisted of thirty-two sentences, in Spanish and English. Participants had to decide whether those sentences were syntactically correct or incorrect (e.g., *Much* soldiers came to the battlefield [‘*Muchos’ soldados acudieron al campo de batalla*]; *Al* perro es perseguido por el gato [*To the* dog is chased by the cat]). Taking in consideration the thirty-two sentences, the Cronbach’s alpha coefficient was 0.50 for the Spanish task and 0.64 for the English task. After dropping 8 items for each language, the Cronbach’s alpha coefficient was 0.61 for the Spanish task and 0.73 for the English task. The final Spanish task consisted of a total of 11 correct and 13 incorrect sentences, while the final English task included 13 correct and 11 incorrect sentences. So, the possible maximum score was 24 in each language, one point for each of the items (pair of sentences) correctly answered.

(c) *Morphological task.* This included eight prefixes and four suffixes, of which students were asked to provide an example of a word with that morpheme (e.g., tri- [meaning: three]; semi- [meaning: half]). Morphemes were different for each language, not the translation of them. The maximum score was 12 in each language, given a point for each correct answer. The Cronbach’s alpha coefficient was 0.60 for the Spanish task and 0.75 for the English task.

(d) *Reading comprehension task*. The Spanish text used (“El ornitorrinco” [“*The platypus*”]) was part of PROLEC-SE-R test (Cuetos et al., 2016). For the English task, we adapted an existing text (“Discovered species”), like the Spanish one in terms of length (English text: 381 words, 16 sentences; Spanish text: 387 words, 15 sentences) and complexity considering the Automated Readability Index (ARI; Senter and Smith, 1967) (English text: 12.17, Spanish text: 11.8). Besides, the English text’s vocabulary used corresponds with a B2 CEFR level in English, according to the Global Scale of English text analyzer of Pearson^1^. After the reading component, participants had to answer 10 multiple-choice questions, both literal (six questions) and inferential (four questions). Participants could score a maximum of ten in each language, one point for each comprehension question correctly answered. The Cronbach’s alpha coefficient was 0.61 for the Spanish task (0.55 reported in the PROLEC-SE-R test) and 0.81 for the English task.

### Procedure

The tasks were presented in a booklet, one for the Spanish and one for the English language. Participants had to complete the booklets on two different days during the month of April. Instructions and one example were presented at the beginning of each task. The completion of each booklet took about an hour. When correcting each task, the number of items with a correct answer was counted, obtaining an overall score for each of the tasks (sum of all the correct items). The research design and procedure were approved by the Ethics Committee for Research of the Principality of Asturias, Spain. It was performed in accordance with the Declaration of Helsinki and the Spanish Law of Personal Data Protection (15/1999 and 3/2018) principles. Before conducting the experimental tasks, parents received information about the study and its objectives and authorized the data collection through signed consent.

### Analysis

Different analyses were conducted with SPSS.24 software package. First, preliminary analyses were performed to assess the normality of the score’s distribution. From the Kolmogorov–Smirnov statistic, we found that five tasks were not normally distributed, so we decided to use non-parametric statistics (even when considering the number of participants some authors approved the use of parametric statistics).

After that, we carried out several Mann–Whitney *U* Tests to check for differences between grades on the tasks. Then, the relationship between tasks’ performance in each language (and between languages) was examined using the Spearmen correlation coefficient. Finally, linear regression analyses were completed to determine if variations in comprehension outcomes could be attributed to variations in the other linguistic tasks.

## Results

### Mann–Whitney *U* Tests

The analysis revealed significant differences between grades in all tasks except the Spanish synonym task, where the difference was close to significance, with better performance in 3rd than in 1st grade. See Table 1.

**TABLE 1:** Linguistic competence in both languages by 1st and 3rd graders.

	Md 1st	Md 3rd	*U*	*z*	*p*-value	*r*
Spanish comprehension	8.00	9.00	293.50	−4.032	0.000	0.48
Spanish vocabulary	19.00	20.00	467.00	−1.941	0.052	0.23
Spanish syntax	21.00	22.00	452.50	−2.125	0.034	0.25
Spanish morphology	9.00	10.00	352.00	−3.271	0.001	0.39
English comprehension	6.00	9.00	157.50	−5.487	0.000	0.65
English vocabulary	17.00	20.00	367.50	−3.066	0.002	0.36
English syntax	16.00	20.00	267.50	−4.195	0.000	0.49
English morphology	6.00	9.00	391.00	−2.802	0.005	0.33

### Spearman Correlations

Spearman correlations were used to explore the strength of relationships between variables. As portrayed in Table 2, a considerable number of interesting correlations were observed. According to questions raised in the study, it is worth noting the positive relationship between the different variables (vocabulary, syntax, and morphology) and English reading comprehension, while in Spanish we solely found a relationship between morphology and reading comprehension. See Table 2.

**TABLE 2:** Correlation matrix among all the tasks for the whole group.

	Spanish vocabulary	Spanish syntax	Spanish morphology	Spanish comprehen	English vocabulary	English syntax	English morphology	English comprehen
Spanish vocabulary		0.130	0.214	0.134	0.237*	0.198	0.118	0.065
		0.275	0.068	0.260	0.043	0.094	0.322	0.585
Spanish syntax			0.376**	0.111	0.195	0.507**	0.243*	0.453**
			0.001	0.352	0.098	0.000	0.039	0.000
Spanish morphology				0.276*	0.347**	0.532**	0.433**	0.462**
				0.018	0.003	0.000	0.000	0.000
Spanish comprehen					0.185	0.282*	0.143	0.538**
					0.117	0.016	0.228	0.000
English vocabulary						0.530**	0.477**	0.398**
						0.000	0.000	0.000
English syntax							0.508**	0.682**
							0.000	0.000
English morphology								0.421**
								0.000

*Correlations for 1st and 3rd grades together.*

*p < 0.05, **p < 0.001.

When the data were split by grade, the fact that no relationship between Spanish reading comprehension and other linguistic tasks was found resulted striking. Meanwhile, in English, correlations were found between reading comprehension and vocabulary, syntax, and morphology in 1st grade, although only with syntax in 3rd grade. See Table 3.

**TABLE 3:** Correlation matrix among all the tasks for each grade.

	Spanish vocabulary	Spanish syntax	Spanish morphology	Spanish comprehen	English vocabulary	English syntax	English morphology	English comprehen
Spanish vocabulary		0.067	0.011	0.094	0.014	−0.043	0.024	−0.186
		0.666	0.942	0.544	0.927	0.781	0.875	0.226
Spanish syntax	0.066		0.345*	0.051	0.132	0.418**	0.379*	0.364*
	0.732		0.022	0.744	0.394	0.005	0.011	0.015
Spanish morphology	0.388*	0.038		0.101	0.251	0.363*	0.476**	0.337*
	0.037	0.846		0.513	0.100	0.016	0.001	0.025
Spanish comprehen	−0.040	−0.069	0.128		0.192	0.171	0.086	0.602**
	0.836	0.721	0.509		0.212	0.266	0.579	0.000
English vocabulary	0.313	0.089	0.078	−0.227		0.418**	0.368*	0.313*
	0.099	0.647	0.688	0.237		0.005	0.014	0.039
English syntax	0.293	0.405*	0.406*	−0.036	0.401*		0.503**	0.601**
	0.123	0.029	0.029	0.853	0.031		0.000	0.000
English morphology	0.105	−0.194	0.201	−0.169	0.548**	0.180		0.325*
	0.587	0.314	0.297	0.382	0.002	0.350		0.031
English comprehen	−0.032	0.368*	0.043	−0.118	0.156	0.450*	0.149	
	0.871	0.050	0.824	0.543	0.420	0.014	0.441	

*Above diagonal for 1st-grade children and under diagonal for 3rd-grade children.*

*p < 0.05, **p < 0.001.

Additionally, taking into account all participants, the relationship between the same tasks in different language (Spanish vocabulary task with English vocabulary task; Spanish syntactic task with English syntactic task; Spanish morphological task with English morphological task; and Spanish reading comprehension task with English reading comprehension task) was also of interest. See Table 2. Finally, considering the different tasks (vocabulary, syntax, and morphology in both languages), in 1st grade a relationship was found between languages for syntax, morphology, and reading comprehension, but only for syntax task in 3rd grade. See Table 3.

### Regression Analysis

Two hierarchical multiple regressions (one for Spanish and one for English languages) were performed to assess the ability of grade and the linguistic measures (vocabulary, morphology, and syntax) to predict reading comprehension outcomes.

With regards to the Spanish reading comprehension, predictors were entered in the following order: grade, Spanish vocabulary, Spanish morphology, Spanish syntax, and English comprehension. The analyses revealed that at Step one, grade contribute significantly to the regression model and accounted for 18.4% of the variance in Spanish reading comprehension, *F*(1,71) = 16.019, *p* = 0.000. At steps 2–4, the independent variables (vocabulary, morphology, and syntax) did not contribute significantly to the regression model, none of the variables was a significant predictor of Spanish reading comprehension. However, step five accounted for an additional 16.2% of variation in Spanish reading comprehension and this change in *R*^2^ was significant, *F*(1,67) = 17.842, *p* = 0.000. However, only the English reading comprehension was a significant predictor of Spanish reading comprehension. See Table 4.

**TABLE 4:** Summary of hierarchical multiple regression analysis for variables predicting the outcome Spanish reading comprehension.

Variable	B	SE	Beta	t	p	*R*	*R* ^2^	Δ*R*^2^
Step 1						0.429	0.184	0.184
Grade	0.698	0.174	0.429	4.002	0.000			
Step 2						0.430	0.185	0.001
Grade	0.709	0.180	0.430	3.939	0.000			
Sp. vocabulary	−0.016	0.059	−0.030	−0.275	0.784			
Step 3						0.477	0.228	0.043
Grade	0.577	0.189	0.355	3.057	0.003			
Sp. vocabulary	−0.023	0.058	−0.043	−0.394	0.695			
Sp. morphology	0.193	0.099	0.224	1.960	0.054			
Step 4						0.478	0.228	0.000
Grade	0.578	0.190	0.355	3.038	0.003			
Sp. vocabulary	−0.022	0.059	−0.040	−0.366	0.716			
Sp. morphology	0.197	0.102	0.228	1.925	0.058			
Sp. syntax	−0.013	0.084	−0.017	−0.152	0.880			
Step 5						0.625	0.391	0.162
Grade	0.084	0.207	0.052	0.409	0.684			
Sp. vocabulary	0.037	0.055	0.068	0.664	0.509			
Sp. morphology	0.118	0.093	0.137	1.263	0.211			
Sp. syntax	−0.118	0.079	−0.157	−1.481	0.143			
Eng. comprehension	0.329	0.078	0.559	4.224	0.000			

As for the English reading comprehension, predictors were entered in this order: grade, English vocabulary, English morphology, English syntax, and Spanish comprehension. Results indicated that at step one, grade contributed significantly to the regression model, accounting for 36% of the variance in English reading comprehension *F*(1,71) = 40.007, *p* = 0.000. After entry of vocabulary at step 2, the contribution (2.7% of variance) to the regression model of this contribution was not significant. The contribution of morphology at step 3 was significant and explained a 3.7% of variance, *F*(1,69) = 4.410, *p* = 0.039. At Step 4 syntax added a 13.7% of explanation of variance, *F*(1,68) = 21.236, *p* = 0.000; and at step 5, final model Spanish comprehension accounted for 8.9% of the variance in English reading comprehension, *F*(1,67) = 17.083, *p* = 0.000. See Table 5.

**TABLE 5:** Summary of hierarchical regression analysis for variables predicting the outcome English reading comprehension.

Variable	B	SE	Beta	t	p	*R*	*R* ^2^	Δ*R*^2^
Step 1						0.600	0.360	0.360
Grade	1.659	0.262	0.600	6.325	0.000			
Step 2						0.622	0.387	0.027
Grade	1.489	0.276	0.539	5.391	0.000			
Eng. vocabulary	0.128	0.073	0.176	1.757	0.083			
Step 3						0.651	0.424	0.037
Grade	1.376	0.275	0.498	5.004	0.000			
Eng. vocabulary	0.050	0.080	0.068	0.620	0.537			
Eng. morphology	0.218	0.104	0.230	2.100	0.039			
Step 4						0.749	0.561	0.137
Grade	0.971	0.257	0.351	3.776	0.000			
Eng. vocabulary	−0.017	0.072	−0.023	−0.231	0.818			
Eng. morphology	0.089	0.095	0.094	0.934	0.353			
Eng. syntax	0.338	0.073	0.470	4.608	0.000			
Step 5						0.806	0.650	0.089
Grade	0.640	0.245	0.232	2.615	0.011			
Eng. vocabulary	−0.005	0.065	−0.007	−0.075	0.941			
Eng. morphology	0.115	0.086	0.122	1.341	0.185			
Eng. syntax	0.277	0.068	0.385	4.097	0.000			
Sp. comprehension	0.576	0.139	0.339	4.133	0.000			

## Discussion

The aim of our study was to explore the Spanish and English reading comprehension in Spanish secondary students attending a bilingual school, as well as their relationship with other linguistic skills. Besides, we wanted to know the contribution of these linguistic skills to reading comprehension. To do this, we carried out several tasks about vocabulary, syntactic and morphological awareness, and reading comprehension in both languages. Our results showed that 3rd graders obtained better results than 1st graders, especially in English. This allowed us to confirm that secondary school students continue developing reading and linguistic skills after primary education, as other authors have already shown (Watson et al., 2012; Álvarez-Cañizo et al., 2020). However, no significant differences between grades were found in Spanish vocabulary (the difference was close to significance). A potential explanation would be that the growth in vocabulary knowledge slows after a certain level (although never ceasing to increase), such as secondary education, and for this reason, we did not find differences between the grades.

Regarding the correlations between reading comprehension and linguistic skills in both languages, and considering both groups together, our results showed that reading comprehension in L1 correlated with morphological awareness. The contribution of morphological awareness to reading comprehension has already been proven, being greater in more advanced grades (Carlisle, 2000). Similarly, 4th-grade Spanish students showed an effect of morphology in reading comprehension (D’Alessio et al., 2019). However, it was reported that morphology helps to infer the significance of words, seemingly indicating that the effect of morphology relates to vocabulary (Crosson and McKeown, 2016). On the other hand, when considering grades separately, there was an absence of relationships between reading comprehension and linguistic abilities in L1. This may seem striking but may be given to the fact that the task’s characteristics do not allow us to catch the influence of these skills in reading comprehension, or perhaps, at certain levels of linguistic proficiency, other skills could be influencing reading comprehension, such as inference making, working memory, previous knowledge, or the ability to monitor the reading activity (Landi and Ryherd, 2017).

As for reading comprehension in L2, when considering 1st and 3rd grades together, reading comprehension correlated with all linguistic tasks (i.e., vocabulary, morphological awareness, and syntactic awareness). However, when grades were considered separately the relationship between reading comprehension and vocabulary and morphological awareness disappeared for 3rd graders. Once again, the relationship between linguistic skills and reading comprehension seems to be determined by age or language proficiency. Vocabulary has been identified as a strong predictor of reading comprehension in English L2 learners (Pasquarella et al., 2012; Farnia and Geva, 2013; van den Bosch et al., 2020), but for native speakers’ vocabulary is decisive for reading comprehension at younger ages (Nation et al., 2007; Ricketts et al., 2007; Hock et al., 2009). As mentioned, we only found a relationship between vocabulary and English reading comprehension for 1st graders, not in 3rd graders in Spanish either. However, the influence of vocabulary on reading comprehension may depend on the text. With regards to morphology, we have already observed that the relationship with reading comprehension varies with age and proficiency level (Carlisle, 2000; Zhang et al., 2020). This way, we could conceive that in the Spanish language as L1, where secondary students demonstrated proficient competencies, the contribution of different skills to reading comprehension differs than in English as L2.

The correlation analysis between languages showed a significant positive relationship in all tasks: reading comprehension, vocabulary, morphological and syntactic awareness when both groups were taken together. This might confirm the linguistic interdependence hypothesis (Verhoeven, 1994). Following this hypothesis, in bilingual learning, language and literacy skills can be transferred from one language (L1) to another (L2), or languages skills have a common basis irrespective of language. It has been seen that this transfer effect also occurs in developing skills, as it is observed in the study of Cisero and Royer (1995). The regression analysis results confirmed the different contributions of linguistic skills to reading comprehension in L1 and L2, a very interesting result. The reading comprehension in Spanish (L1) is explained by English reading comprehension. Morphology correlated with reading comprehension, but the regression analysis indicated that the main predictor of Spanish reading comprehension was English reading comprehension, after controlling the effect of the grade. As previously stated, morphological awareness is a skill that continues to develop throughout the school years (Casalis and Louis-Alexandre, 2000), along with reading expertise (Rastle, 2019). In addition, several studies demonstrated its relationship with reading comprehension, since it contributes significantly to knowing the meaning of words, thus favoring the understanding of the text (e.g., Deacon and Kirby, 2004; Cain and Oakhill, 2006; D’Alessio et al., 2019; Zhang et al., 2020; Li et al., 2021). However, the study of Zhang et al. (2020) supports that the contribution of morphological awareness depends on language proficiency. It is possible that L1 students have reached a sufficient level of vocabulary, syntactic and morphological awareness, so that they no longer influence reading comprehension, although these skills continue to develop at these ages, as we have seen in our results and in previous studies (Casalis and Louis-Alexandre, 2000; Hock et al., 2009).

Regarding English (L2), reading comprehension was explained by grade, syntax, and Spanish reading comprehension. According to this, it should be highlighted the importance of language exposure and competence, as variance of English reading comprehension is determined by grade. As far as syntax awareness is concerned, it was supposed to be an important predictor of reading, helping to anticipate words and make inferences (Bishop and Snowling, 2004), but the role of syntax was different for L1 and L2. The differences between English and Spanish syntax (Klavans, 1985) could make it a determining variable in L2 reading comprehension. In addition, Spanish reading comprehension also appeared to be a good predictor of English reading comprehension, supporting the interdependence hypothesis between languages (Verhoeven, 1994). In addition, it could be hypothesized that other variables, related to reading comprehension, could be influencing reading comprehension in both Spanish and English; as Cummins (1979) considered, there could be some underlying cognitive or academic proficiency common across languages, which eases the transfer of cognitive, academic, and literacy-related skills.

In closing, this study is a pioneer in the examination of reading comprehension in Spanish L1 as in English L2. It can be concluded that reading comprehension along with other linguistic skills continue developing well into secondary school, both in L1 and L2, with a better performance in L1. Besides, we can support a transfer or interdependence effect between languages as previously proved by different authors, such as the Linguistic Interdependence Hypothesis (Verhoeven, 1994). Finally, it seems that the language proficiency in Spanish (L1) and English (L2), given the differences in exposure to them, determines the linguistics skills related to reading comprehension, as previously proven by other authors (Jiang, 2011; Edele and Stanat, 2016).

## Implications

The findings in our study allow us to highlight the importance of certain abilities for reading comprehension, as well as the need to increase exposure to a second language to facilitate the development of different language skills, which ultimately have an impact on reading comprehension. Regarding English reading comprehension, specific attention should be given to syntactic awareness, bearing in mind its important contribution to reading comprehension.

## Limitations

Despite the considerable results of this study, we would like to mention some limitations or noteworthy aspects to be included in future studies. Results seem to help us understand the contribution of certain linguistic skills to reading comprehension in L1 and L2, but results should be interpreted with caution due to the relatively small groups, limited range of grades, and the near ceiling effect in some tasks. It could be interesting to include or explore the contribution to reading comprehension of some abilities such as working memory, previous knowledge, or the ability to make inferences while reading. Furthermore, it could be interesting to expand the sample with students from other high school grades, in order to comprehend the development of reading comprehension in L2 students, taking into account that our sample was not very sizeable. The use of larger sample sizes could also allow the performance of mediation analysis, to study indirect effects of certain skills. Besides, the tasks used to assess the different linguistics skills could be complemented, as making decisions based on a single score is generally a poor practice. However, it is necessary to find a balance between cost-benefit, especially when it comes to working with children. For example, in the vocabulary tasks, it could be interesting to include an expressive vocabulary task (e.g., picture naming), rather than just a comprehensive task such as semantic judgment.

## Data Availability Statement

The raw data supporting the conclusions of this article will be made available by the authors, without undue reservation.

## Ethics Statement

The studies involving human participants were reviewed and approved by Ethics Committee for Research of the Principality of Asturias, Spain. Written informed consent to participate in this study was provided by the participants’ legal guardian/next of kin.

## Author Contributions

PS-C, MÁ-C, and EC carried out the research design and preparation of the materials. EC performed the data collection and analysis. All authors contributed to the preparation of the manuscript.

## Conflict of Interest

The authors declare that the research was conducted in the absence of any commercial or financial relationships that could be construed as a potential conflict of interest.

## Publisher’s Note

All claims expressed in this article are solely those of the authors and do not necessarily represent those of their affiliated organizations, or those of the publisher, the editors and the reviewers. Any product that may be evaluated in this article, or claim that may be made by its manufacturer, is not guaranteed or endorsed by the publisher.

## Footnotes

1
https://www.pearson.com/english/about-us/global-scale-of-english.html
